# Immunopathology of metabolic syndrome: effects on gut microbiota and antibiotic resistance

**DOI:** 10.3389/fimmu.2026.1926461

**Published:** 2026-10-30

**Authors:** Navid Namadian, Valentyn Oksenych, Zahra Asadi, Niloofar Kiaheyrati, Hamed Tahmasebi

**Affiliations:** 1 Department of Bacteriology, Pasteur Institute of Iran, Tehran, Iran; 2 Division of Microbiology, Department of Pathobiology, School of Public Health, Tehran University of Medical Sciences, Tehran, Iran; 3 Faculty of Medicine, University of Bergen, Bergen, Norway; 4 Department of Microbiology, Danylo Halytsky Lviv National Medical University, Lviv, Ukraine; 5 Oslo Bioconsulting, Oslo, Norway; 6 School of Medicine, Shahroud University of Medical Sciences, Shahroud, Iran

**Keywords:** antibiotic resistance, dysbiosis, gut microbiota, immunopathogenesis, inflammation, metabolic syndrome, resistome

## Abstract

The cluster of related metabolically derived disorders that confer increased risk of developing type 2 diabetes mellitus (T2D) and cardiovascular disease (CVD) is termed the Metabolic syndrome (MetS). Despite two decades of growing evidence on MetS pathogenesis, the immunopathological links between gut microbiota (GM) dysbiosis and metabolic derangements remain only partially elucidated. This review focuses on the complex nexus among MetS, GM composition and function, and the emerging antibiotic-resistance predicament. The gut commensal microbiome-defined as approximately 10^14^ microorganisms that have been termed endocrine organs with key roles beyond gastrointestinal homeostasis, such as immune-mediated host inflammatory and metabolic regulation and barrier homeostasis- undergoes dysbiosis in MetS, characterized by marked losses of beneficial short-chain fatty acid (SCFA)-producing bacteria and overgrowth of pathobiont *Proteobacteria*, such as those bearing lipopolysaccharide. Increased gut permeability and Toll-like receptor 4 (TLR-4) activation, driven by increased luminal products, are termed metabolic endotoxemia and form the immunopathological bridge between gut dysbiosis and systemic low-grade inflammation and insulin resistance. Disturbances in microbial-mediated host metabolism of bile acids, tryptophan catabolism, and the regulatory T cell/T helper 17 axis further promote inflammation in adipose tissue, liver, and skeletal muscle. Antibiotic-driven dysbiosis, which reduces diversity and increases antibiotic-resistant gene elements within the host gut resistome, exacerbates metabolic abnormalities and is a key focus for developing personalized treatment approaches.

## Graphical Abstract

**Figure:**
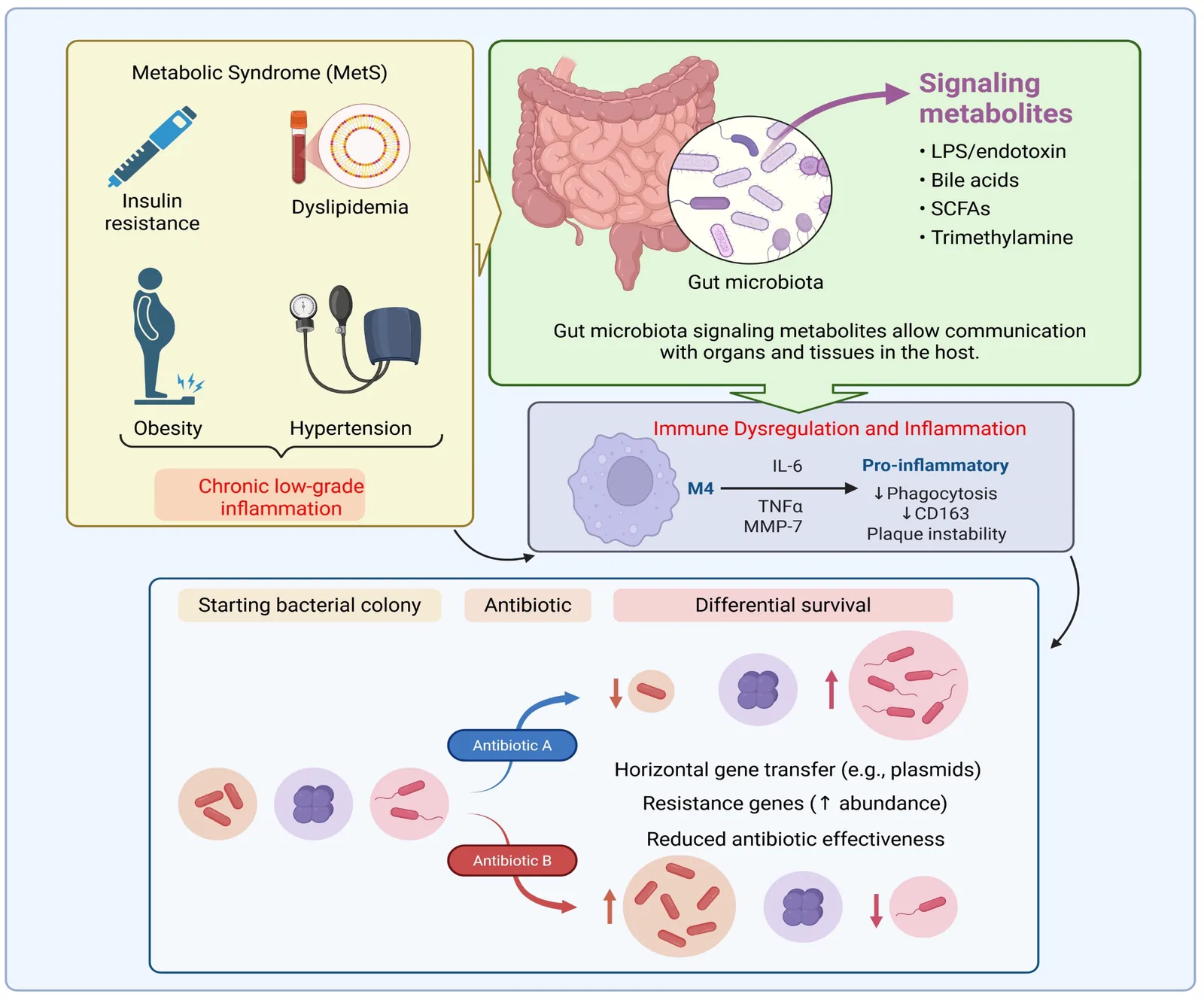

## 1 Introduction

Metabolic syndrome (MetS) is undoubtedly one of the greatest public health burdens of the twenty-first century, affecting over a quarter of the adult population worldwide and placing a considerable burden on health systems (1). MetS combines cardiometabolic risk factors, including abdominal obesity, elevated fasting glucose, dyslipidemia, and hypertension, that promote the development of type 2 diabetes mellitus (T2D) and atherosclerotic cardiovascular disease (2, 3). Numerous investigations have defined the bacterial, dietary, and lifestyle factors leading to MetS and T2D in human hosts; however, recent developments have provided further insight into an emerging role of the human gut microbiome as an ‘environmental epigenetic’ regulator of host health, through mechanisms such as modulation of carbohydrate fermentation, vitamin and bile acid metabolism, and interaction with the developing immune system. The human gut microbiome comprises a vast diversity of bacteria, archaea, viruses, protists, and fungi; the bacterial component contains>1000 species and encodes a metagenome thousands of times larger than the human genome (4–6).

Crosstalk between MetS and the gut microbiota (GM) via immunopathological pathways: Metabolic deregulation and inflammation in MetS are intricately linked to GM composition and function in a complex, bidirectional relationship (7). A MetS-like state creates conditions that select for dysbiotic microbial communities by enriching the gut environment with gluco- and lipotoxic metabolites and inducing low-grade mucosal inflammation (8). Conversely, specific compositional and functional phenotypes of GM directly induce the hallmarks of MetS (increased energy intake, disrupted epithelial barrier, immune activation) through multiple mechanisms involving increased energy extraction, deconjugation of bile acids and choline, translocation of bacterial LPS and other microbial metabolites, disruption of the gut mucosal barrier, and low-grade immune cell activation and expansion in the gut, adipose tissue, pancreas, and the liver (9–11). This resulted in a pathological positive feedback loop of dysbiosis-induced inflammation, which exacerbates the expression of MetS; importantly, the immunopathological component seems critical in MetS, which in fact appears to be an inflammatory “syndrome”, best referred to as “meta-inflammation”, a syndrome of compromised immune regulation involving the activation of innate and adaptive immune cells in metabolic tissues (12).

High-throughput sequencing methods (16S ribosomal RNA gene amplicon sequencing and shotgun metagenomics) have extensively characterized changes in the gut microbiome associated with metabolic disease, with the recognition of reproducible dysbiotic signatures, including decreased diversity, losses of the beneficial members of the Firmicutes, including *Faecalibacterium prausnitzii* and *Roseburia intestinalis*, and expansions of Gram-negative *Proteobacteria*, including Enterobacteriaceae (13, 14). These compositional changes also altered microbial functional capacity, with decreased short-chain fatty acids (SCFAs) and increased lipopolysaccharide (LPS) biosynthesis. This rapid accumulation of knowledge has prompted extensive research into bacterial molecular components and host receptor pathways engaged by the altered GM that drive local and systemic inflammatory processes and regulate host metabolism (15, 16).

Another dimension of the microbiota-MetS link is antibiotic resistance. Since their widespread introduction, antibiotic use in hospital and agricultural settings has dramatically perturbed human microbial ecosystems, often with irreversible detrimental consequences for host metabolism (17). After exposure, commensal bacteria can remain depleted for months to years after the agent is removed. In particular, antibiotic therapy creates selective pressure for resistance gene acquisition among beneficial bacteria, thereby expanding resistant bacteria and promoting the transfer of resistance genes within the gut resistome (18, 19). In the already dysbiotic state of MetS, adding antibiotics may further disrupt the balance between beneficial and harmful bacteria, compromise gut barrier integrity, and exacerbate inflammatory signaling (20). Nevertheless, antibiotic resistance may also affect host metabolism beyond the antibiotic’s inefficacy in treating the illness, as resistance factors may influence host innate immune and metabolic pathways.

This review aims to provide a critical overview on immunopathological mediators contributing to MetS, disturbances of the microbial community, and antibiotic resistance, through a thorough assessment of data derived from human studies, animal models and *in-vitro* analysis; it intends to re-evaluate the reciprocal influences among these factors critically; it will also assess methodologies for the reconciliation of contradictory results, and discuss perspectives and limitations for therapeutic strategies targeting the microbial environment, with the final goal to offer deeper insight into host-microbe reciprocal immune pathways influencing metabolic disease pathogenesis and a possible scenario to focus for further therapeutic strategies targeting the specific disturbance at a precision level with the scope to restore the host-microbe and metabolic balance.

## 2 Methodology

### 2.1 Search strategy and databases

We conducted a comprehensive review of relevant research using electronic databases, including MEDLINE/PubMed, Embase, Scopus, the Cochrane Library, and Web of Science. We ran the final search on 15^th^ June 2026. We combined Medical Subject Headings and free-text words, which covered three themes, namely (1) MetS and related metabolic disorders (2); Gut microbial flora, dysbiosis and microbial metabolites (3); Immuno-inflammation and antibiotic resistance. Search strategies used relevant Medical Subject Headings or terms and free text words: ‘MetS’, ‘Insulin resistance’, ‘Fatness’, ‘Type 2 diabetes’, ‘Gut microbiome’, ‘Intestinal microbiome’, ‘Dysbiosis’, ‘Short chain fatty acids’, ‘Lipoprotein’, ‘Metabolic endotoxemia’, ‘TLR-4’, ‘Inflammation’, ‘Cytokines’, ‘T regulatory cells’, ‘T helper 17 cells’, ‘Antibiotic resistance’, ‘Resistome’, ‘Antibacterial resistance genes’, and ‘Fecal microbiota transplantation’. We searched these terms using both logical combinations and truncation, and we also searched again for references found in review systems.

### 2.2 Language and publication-type restrictions

We restricted the search to English-language, peer-reviewed literature. No geographical restrictions were used. For publication types, we considered primary research articles the main body of evidence, with preference given to randomized controlled trials, prospective and retrospective cohort studies, case-control studies, and cross-sectional observations. We included systematic and narrative reviews and meta-analysis if they covered key aspects of both topics. Conference abstracts, commentaries, and editorials were not accepted unless they were the only source reporting a novel mechanism of significance. We generally excluded literature earlier than 2000 unless it was the defining investigation establishing MetS etiology and pathophysiology or the first demonstration of metabolic endotoxemia or gut microbiome transfer compared with its naïve counterpart.

### 2.3 Criteria for including human, animal, and *in vitro* evidence

We prioritized available evidence from human studies, including randomized controlled trials, well-designed prospective cohort studies, and controlled intervention studies with relevant metabolic markers. Human studies must have used validated diagnostic criteria for MetS, such as the IDF, NCEP-ATP III, or Harmonized criteria, and studies that characterized human microbiomes using metagenomic sequencing, metabolomics, or immunophenotyping were considered more mechanistically informative. We also sought *in vivo* animal studies using physiologically relevant MetS models, including high-fat diet-induced obesity, genetic models of leptin deficiency or resistance, or inbred mouse strains in which a high-fat diet or insulin resistance induced MetS. Experiments using germ-free mice, antibiotic-treated mice, or fecal microbiota transplantation (FMT) models between colonized, obese, and lean host animals were essential to establish causality. We also included *in vitro* studies that shed light on cellular or molecular mechanisms, especially those investigating th*e* effects of microbial metabolites or PAMPs on immune cells, epithelial barrier function, or insulin signaling pathways. Interpret *in vitro* studies with caution because of limited translatability; they may support only mechanistic hypotheses.

### 2.4 Approach to prioritizing primary studies, systematic reviews, and recent literature

The evidence hierarchy framework prioritizes primary, high-quality studies and systematic reviews. This framework requires systematic reviews and meta-analysis to analyze the magnitude of the reported effects and whether they were consistent across primary studies. However, when systematic reviews produced conflicting results, we repurposed the original studies to identify potential sources of heterogeneity, including sample size, microbiota assessment methods, metabolic endpoints, and intervention protocols. We also emphasized studies from the last five years to reflect the current state of affairs and recent developments, especially in multi-omics and the characteristics of the gut resistome. Moreover, we highlighted key papers that introduced new ideas, such as the hypothesis of metabolic endotoxemia, the establishment of the transfer of microbial metabolism, and the discovery of *Akkermansia muciniphila* as a beneficial microbe.

### 2.5 Method used to assess conflicting evidence

The systematic framework assessed conflicting evidence by evaluating research design, sample size, methodological rigor, biological plausibility, and consistency with other evidence. When animal experiments produced contradictory results, such as different metabolism in Toll-like receptor 4 (TLR-4) knockout mice across different research laboratories, the variables assessed included the genetic background of the animals used in the experiments, diet, duration of the process, sex of the animals, and the features of the microbiota composition of the animals involved. For the human population, we considered issues already identified as sources of inconsistency, such as metabolism, dietary habits, medications used, and geographical features. We also considered publication bias and the file drawer effect, especially in areas where positive results dominate. If the evidence was truly contradictory, we noted it and explained its impact.

## 3 MetS: definition and immunopathological foundations

### 3.1 Diagnostic criteria and global burden

MetS is characterized by the co-occurrence of multiple cardiometabolic risk factors that synergistically increase the risk of progressing to T2D and CVD (21). Most current MetS diagnostic frameworks follow the International Diabetes Federation (IDF) criteria, which require concurrent central obesity with at least 2 other metabolic abnormalities: increased triglycerides, reduced HDL-C, raised BP, and/or elevated fasting plasma glucose (22).

Consensus-based initiatives have also proposed alternative definitions, including the National Cholesterol Education Program Adult Treatment Panel III (ATP III) and, more recently, the Harmonized Criteria, both of which use analogous but slightly different cut-offs for individual components based on ethnicity (21, 23). Despite methodological differences, these classification systems share an overarching pathophysiological commonality: MetS reflects systemic insulin resistance (24).

Widespread epidemiological observation and growing mechanistic insight into its molecular pathology show an alarming worldwide escalation in MetS prevalence in recent decades, directly attributable to the exponential rise in consumption of energy-dense/nutrient-poor foods alongside increasingly sedentary lifestyles. Understanding this multifactorial syndrome from a translational perspective is therefore both an important clinical challenge and a critical public health imperative (25).

### 3.2 Low-grade chronic inflammation as a central pathophysiological feature

Recognizing chronic low-grade inflammation as a key underlying pathological process marked a major paradigmatic shift in MetS research (26). Inflammatory conditions typically involve persistent activation of innate and adaptive immune cell subsets. Researchers have extensively documented this across organs such as the liver, skeletal muscle, and pancreatic islets under MetS-associated metabolic stressors (26, 27).

Histological examination of adipose tissue from obese people revealed significant macrophage infiltration into fat deposits; these infiltrating cells predominantly exhibit the ‘classically activated’ pro-inflammatory M1 subset [29] with elevated levels of key inflammatory mediators, e.g., TNF-alpha, IL-6, and IL-1beta (28, 29). These factors impair insulin signaling by enhancing serine phosphorylation of IRS-1, activating the JNK and IKK (IKB kinase) pathways, and upregulating SOCS family proteins (27, 30). Adipose tissue-derived inflammatory stimuli attract both CD8+ cytotoxic T-cell subsets and T-helper 1 (Th1)-pro-inflammatory effector cells, which promote early macrophage infiltration and may help sustain the local inflammatory state once established (31, 32).

### 3.3 The gut as an immunometabolic interface

As a key immunometabolic interface, the gut tract enables interactions among intestinal nutrition, microbial signals, and immune events that ultimately mediate whole-body energy balance. The intestinal epithelium, a single-layer columnar epithelium, acts as a selective barrier, allowing absorption of essential nutrients while restricting translocation of potentially dangerous products from the intestinal lumen, including bacteria, toxins, and antigens. Goblet cells produce mucus, Paneth cells synthesize and release AMPs, and tight junctions between epithelial cells restrict paracellular passage (33, 34).

A complex network of dendritic cells, macrophages, innate lymphoid cells, and adaptive lymphocytes (dendritic cells, Macrophages, ILCs, and T and B cells) resides within the Lamina propria, monitors the intestinal lumen at all times, and mounts immune responses. In MetS, the structural integrity of the intestinal immunometabolic interface is impaired due to intestinal permeability and entry of bacterial products into the blood, termed ‘ metabolic endotoxemia,’ which is considered an important mechanistic linkage between gut microbial changes and inflammation, because LPS can stimulate TLR4 signaling, which in blood cells and metabolic tissues results in systemic inflammatory cascades and induction of insulin resistance (33, 35) (**Figure 1****).**

**Figure 1:**
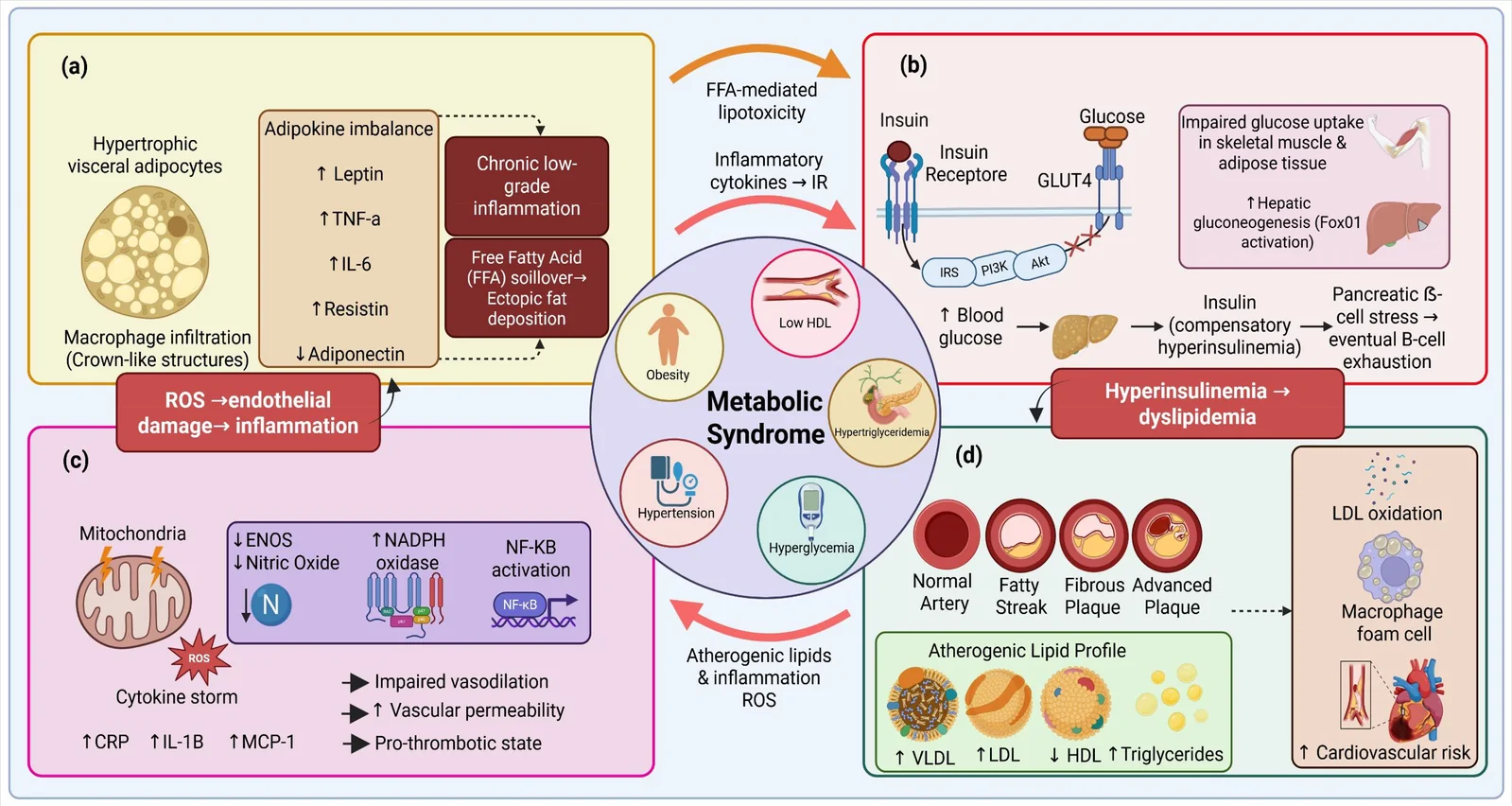
Metabolic syndrome links adipose dysfunction, insulin resistance, dyslipidemia, oxidative stress, and atherosclerosis. In **(a)**, hypertrophic visceral adipocytes recruit macrophages, forming crown-like structures and altering adipokines (↑leptin, TNF-α, IL-6, resistin; ↓adiponectin), driving chronic low-grade inflammation. Adipose lipid overflow releases free fatty acids, causing ectopic fat deposition and further inflammation. In **(b)**, inflammatory cytokines impair insulin receptor–IRS–PI3K–Akt signaling, reducing GLUT4-mediated glucose uptake in muscle and adipose tissue while increasing FoxO1-dependent hepatic gluconeogenesis. Hyperglycemia induces compensatory hyperinsulinemia, promoting dyslipidemia and pancreatic β-cell stress, exhaustion, and failure. Metabolic syndrome components generate atherogenic lipids and ROS. In **(c)**, mitochondrial ROS and NADPH oxidase reduce eNOS/nitric oxide, impair vasodilation, activate NF-κB, and trigger CRP, IL-1β, MCP-1, vascular permeability, and a prothrombotic state. In **(d)**, ↑VLDL, LDL, and triglycerides plus ↓HDL foster LDL oxidation, macrophage foam cells, fatty streaks, fibrous and advanced plaques, culminating in increased cardiovascular risk. These pathways promote vascular injury, thrombosis, and clinical events. Created with BioRender.com.

## 4 Gut microbiota composition and function in metabolic homeostasis

### 4.1 The healthy gut microbiome: diversity, stability, and functional capacity

Our healthy adult gut microbiome is defined by two major phyla, the Firmicutes and Bacteroidetes, which make up roughly 90% of all bacteria. *Actinobacteria*, *Proteobacteria*, and *Verrucomicrobia* make up the remainder of gut bacteria and, though numerically smaller, can be critically important. Individual variation in microbiome composition is tremendous; however, many bacterial groups can fill the same metabolic niche. Maintaining gut microbiome stability involves ecological interactions such as nutrient competition, cross-feeding, and the production of antibiotics known as bacteriocins, all of which limit opportunistic bacterial proliferation (35–39).

Healthy GM is metabolically important and carries out processes such as polysaccharide digestion, short-chain fatty acid synthesis, amino acid fermentation, and bile acid conversion. Gut microbes use these metabolic capabilities to produce many small molecules that profoundly influence host physiology, including appetite regulation and metabolic expenditure, and mediate many effects on host immune cells. Gut microbes with higher functional or even taxonomic diversity seem better equipped to resist environmental change and withstand fluctuations in dietary intake, antibiotic administration, or pathogen invasion (37–40) (**Figure 2****).**

**Figure 2:**
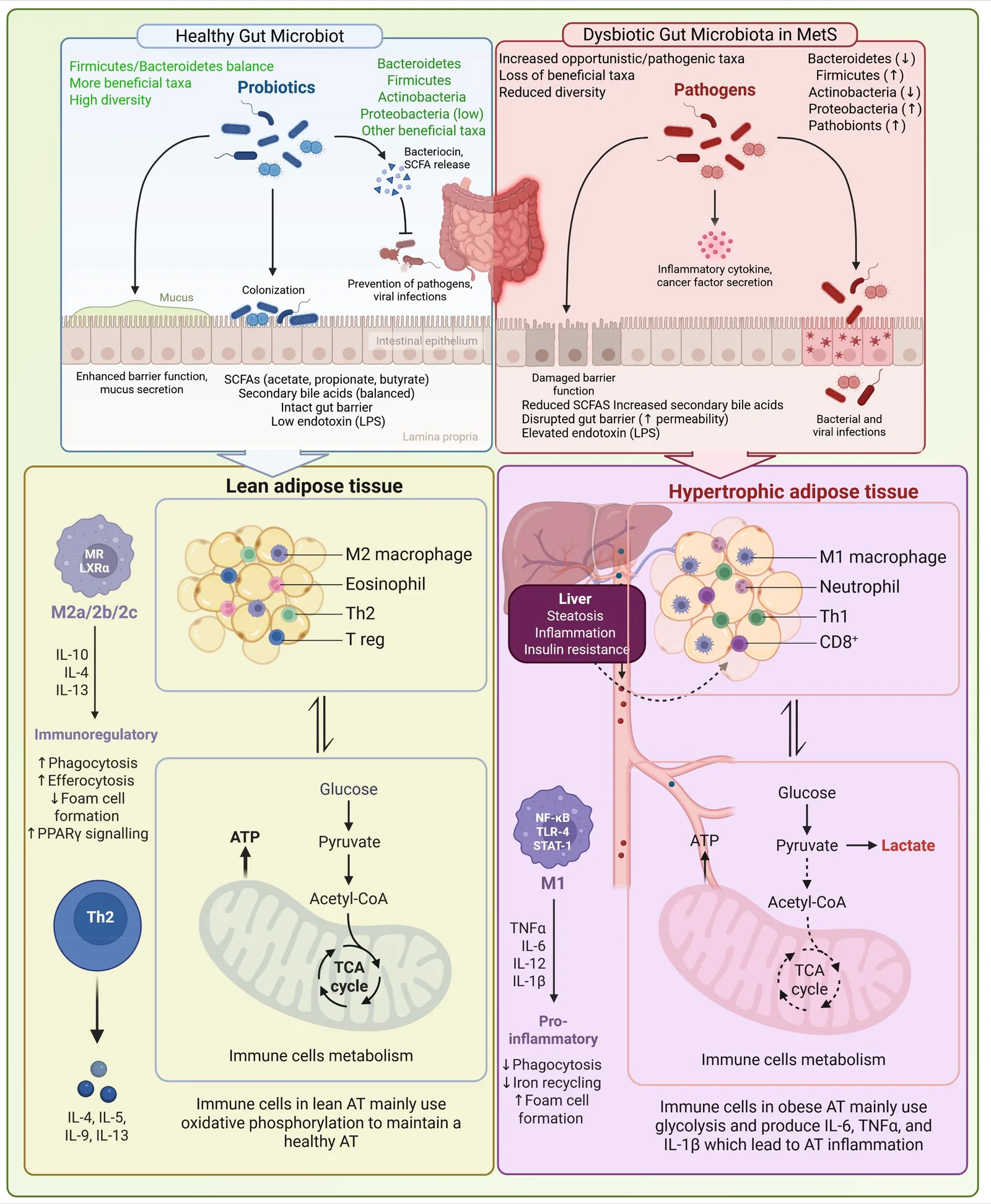
A healthy microbiota maintains the Firmicutes/Bacteroidetes balance, beneficial taxa, and diversity. Probiotics release bacteriocins and SCFAs, colonize mucus and epithelium, and prevent pathogens and viral infection. SCFAs (acetate, propionate, butyrate), balanced secondary bile acids, mucus, and low LPS preserve barrier integrity. Obesity-associated dysbiosis increases pathogens, Firmicutes, Proteobacteria, pathobionts, secondary bile acids, and permeability, while reducing diversity, Bacteroidetes, Actinobacteria, beneficial taxa, and SCFAs. Pathogens promote infection and inflammatory/cancer-factor secretion; leaky barriers and LPS link gut dysfunction to adipose and hepatic steatosis, inflammation, and insulin resistance. Lean adipose contains MR/LXRα+ M2a/2b/2c macrophages, eosinophils, Th2 cells, and Tregs. IL-4, IL-5, IL-9, IL-10, and IL-13 enhance phagocytosis, efferocytosis, PPARγ, and glucose–TCA–ATP OXPHOS while limiting foam cells. Hypertrophic obese adipose tissue instead recruits M1 macrophages, neutrophils, Th1 cells, and CD8+ T cells. NF-κB–TLR4–STAT1 signaling induces TNF-α, IL-6, IL-12, and IL-1β, reducing phagocytosis and iron recycling, increasing foam cells, and favoring glycolysis, lactate production, and TCA suppression, driving adipose inflammation. Created with BioRender.com.

### 4.2 Short-chain fatty acids as critical microbial metabolites

The main metabolic end-products of anaerobic microbial fermentation of dietary fiber are acetate, butyrate, and propionate, and SCFAs represent a significant mechanism by which the microbiota alter host metabolism and immunology (41, 42). Epithelial cells lining the colon use butyrate as a preferred energy source, contributing up to sixty to seventy percent of their energy needs and supporting epithelial proliferation, differentiation, and barrier function. The impact of butyrate extends to epithelial proliferation where, in addition to providing trophic support to the epithelium, the SCFA also acts as a signaling molecule to activate G protein-coupled receptors including GPR43 and GPR109A in gut immune and enteroendocrine cells and adipocytes; these receptors then induce signal cascades that alter inflammation, insulin sensitivity, and energy homeostasis (40, 43–45).

Additionally, butyrate can inhibit histone deacetylases (HDACs), altering immune cell gene expression to augment anti-inflammatory profiles, boost regulatory T cell function, and dampen pro-inflammatory cytokine production (46, 47). Propionate, derived mainly from *Bacteroidetes* members, undergoes substantial hepatic metabolism and inhibits both cholesterol synthesis and gluconeogenesis, whereas Acetate enters systemic circulation and appears to affect appetite regulation at a central level as well as promoting lipogenesis and immunity. Together, the end products of microbial anaerobic fermentation play a critical role in integrating microbiota metabolism into the host, and altered production of these SCFAs has consistently correlated with MetS pathogenesis (40, 48).

### 4.3 Bile acid metabolism and microbial biotransformation

Bile acids, originally cholesterol-derived messengers secreted by the liver into the gut lumen as aids for lipid digestion and absorption, are substantially biotransformed by gut microbes. Primary bile acids such as cholic acid and chenodeoxycholic acid that are normally conjugated to glycine or taurine during secretion undergo desulfonation and dehydroxylation by bacterial bile salt hydrolases and 7-alpha-dehydroxylases that convert them into secondary bile acids, deoxycholic acid and lithocholic acid. This microbial modification dramatically increases the complexity of bile acid signal diversity and confers upon the gut hormone functions for the orchestration of metabolism via activation of farnesoid X receptor (FXR) and Takeda G-protein-coupled receptor 5 (TGR5) (49–51).

In the intestine and liver, FXR regulates intestinal and hepatic gluconeogenesis, lipid metabolism, and inflammation; in the liver, FXR activation also activates the nuclear receptor Rev-erbalpha, which inhibits clock genes such as BMAL1. TGR5 activation in brown adipose tissue and skeletal muscle stimulates energy expenditure and inhibits inflammation. GM then acts as a metabolic gatekeeper for bile acid pool signaling. Microbial transformation of bile acids has increasingly been linked to insulin resistance, liver steatosis, and generalized inflammation in MetS (51–54).

### 4.4 Tryptophan metabolism and the aryl hydrocarbon receptor pathway

Indoleamine and its precursors have been extensively reported to act on immune cells and the CNS. Indispensable aromatic amino acid obtained through diet that becomes the substrate for many host and bacterial metabolic pathways that provide active substances (41, 47). Host-specific enzymes, indoleamine-2,3-dioxygenase and tryptophan-2,3-dioxygenase, transform tryptophan to kynurenine and downstream metabolites. Kynurenine and its derivatives regulate immune cell development and function, neurotransmission, and can induce tolerogenic dendritic cells (55, 56).

Meanwhile, tryptophanase and its analogous bacterial enzyme from commensal gut bacteria produce indole and its downstream compounds like indole-3-aldehyde, indole-3-propionic acid, and indole-3-acetic acid from the host’s tryptophan. Bacterial indole derivatives act as ligands of the aryl hydrocarbon receptor (AhR), a ligand-inducible transcriptional factor present in colon epithelial cells, immune cells, and hepatocytes (57). AhR agonists, such as bacterial tryptophan derivatives, stimulate IL-22 expression from group 3 innate lymphoid cells, strengthen epithelial barrier integrity, enhance antimicrobial peptide secretion, and modulate T-cell differentiation to favor Treg. Therefore, the tryptophan-AhR system links microbial and host immunity; disruption of bacterial tryptophan metabolism increases intestinal permeability, triggers an overreactive inflammatory response, and drives metabolic dysfunction associated with obesity and MetS (55, 58, 59) (**Table 1****).**

**Table 1:** Characteristics of gut microbiota alterations in metabolic syndrome.

Microbial feature	Change in MetS	Representative taxa/groups	Functional change	Relevance to MetS	Ref.
Microbial diversity	↓ Reduced	Overall gut microbiota	Loss of community richness and functional redundancy; reduced ecosystem resilience and recovery after dietary or antibiotic perturbation.	Greater dysbiosis, altered microbial metabolites, weaker colonization resistance, and greater susceptibility to metabolic and inflammatory disturbances.	(60)
Firmicutes/Bacteroidetes ratio	Often ↑/altered	*Firmicutes* and *Bacteroidetes*	Shifts in relative abundance may modify dietary carbohydrate fermentation and energy harvest; the ratio depends on cohort, diet, and medication, rather than serving as a universal diagnostic marker.	Potentially greater caloric harvest, adiposity, and insulin resistance when accompanied by broader loss of metabolic diversity.	(61)
SCFA-producing bacteria	↓ Reduced	*Faecalibacterium prausnitzii*, *Roseburia* spp., *Eubacterium* spp.	Reduced fermentation of complex carbohydrates lowers butyrate and other SCFAs that support colonocyte metabolism, epithelial integrity, and anti-inflammatory signaling.	Higher intestinal permeability, impaired glucose regulation, and chronic low-grade inflammation.	(62)
Mucin-associated beneficial bacteria	↓ Reduced	*Akkermansia muciniphila*	Reduced mucus-layer maintenance and altered host-microbe interaction at the intestinal surface; may weaken tight-junction integrity and epithelial signaling.	Greater barrier dysfunction, LPS translocation, metabolic endotoxemia, and impaired insulin sensitivity.	(63)
Opportunistic pathogens (pathobionts)	↑ Increased	*Escherichia coli*, *Shigella* spp., *Proteobacteria*/*Enterobacteriaceae*	Expansion of inflammatory Gram-negative organisms increases LPS exposure and may reduce ecological colonization resistance.	Enhanced endotoxemia, innate immune activation, adipose inflammation, and metabolic deterioration.	(64)
Inflammation-associated bacteria	↑ Increased	*Collinsella* spp., *Prevotella copri*, *Bacteroides vulgatus*	Changes in microbial metabolism and host-microbe signaling; some taxa are linked to altered intestinal inflammatory tone and nutrient utilization.	Associated with insulin resistance, dyslipidemia, and persistent low-grade inflammation.	(65)
Butyrate production capacity	↓ Reduced	Multiple butyrate-producing Firmicutes	Lower butyrate availability reduces fuel for colonocytes and weakens pathways supporting epithelial oxygen balance, mucus production, and tight-junction function.	Loss of barrier integrity may amplify endotoxemia and systemic inflammation.	(66)
LPS-producing bacteria	↑ Increased	Gram-negative *Proteobacteria*/*Enterobacteriaceae*	Greater endotoxin production promotes TLR4-dependent innate signaling and downstream NF-κB-mediated inflammatory responses.	Metabolic endotoxemia, hepatic and adipose inflammation, and worsening insulin resistance.	(67)

MetS, metabolic syndrome; SCFAs, short-chain fatty acids; LPS, lipopolysaccharide; TLR4, Toll-like receptor 4; NF-κB, nuclear factor kappa B.

## 5 Dysbiosis in MetS: compositional and functional alterations

### 5.1 Reduced microbial diversity and loss of beneficial taxa

As shown in **Figure 3**, GM composition differs in MetS individuals compared with healthy controls and other metabolic conditions, with a consistent loss of microbial diversity, as assessed by alpha diversity indices. This loss largely reflects the loss of functionally relevant taxa, such as obligate anaerobes in the phylum Firmicutes, which specialize in complex carbohydrate fermentation and short-chain fatty acid production. *Faecalibacterium prausnitzii* is among the most abundant butyrate-producing bacteria of the human colon and has been identified as a biomarker of a healthy gut (8, 68, 69).

**Figure 3:**
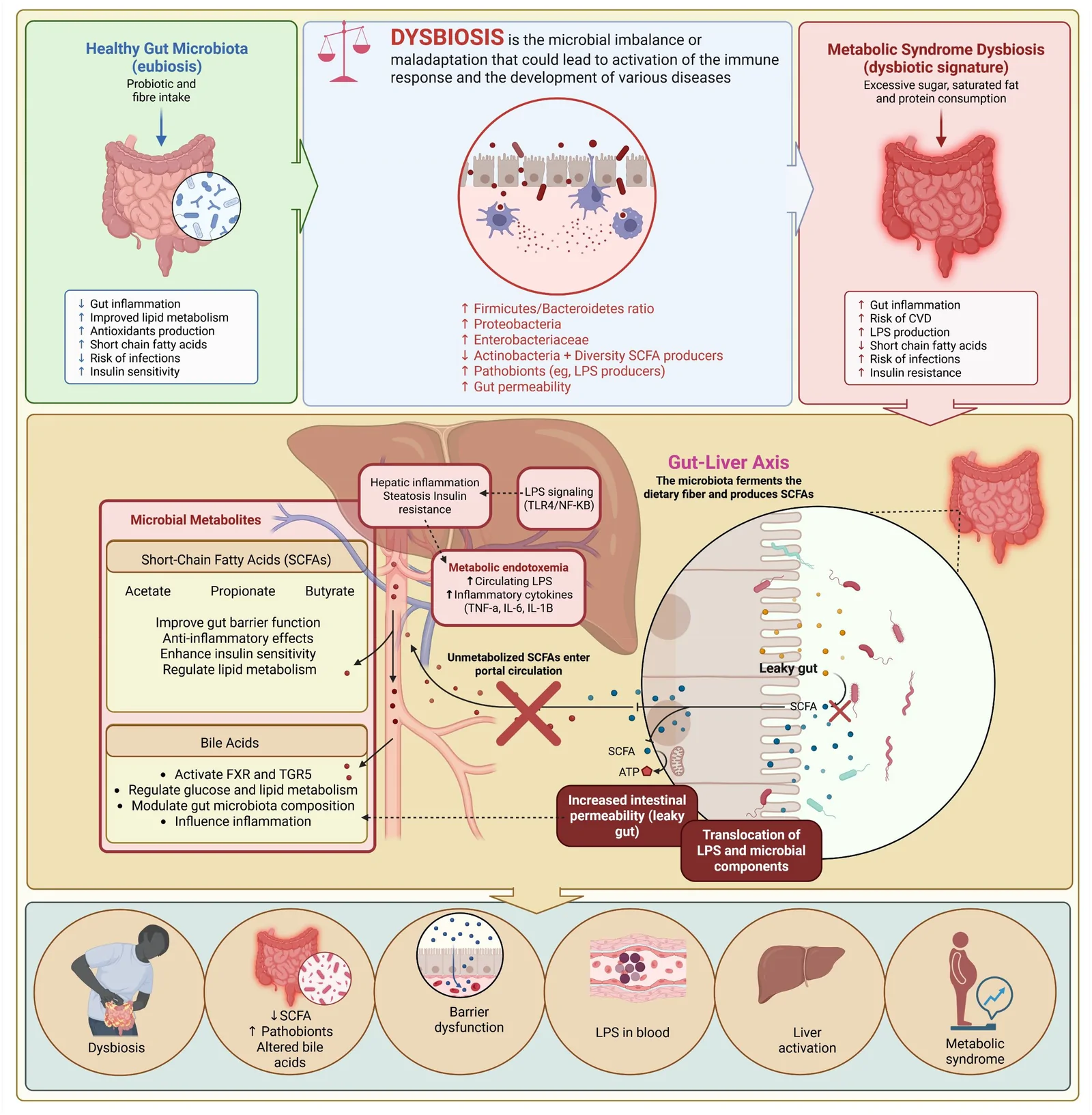
Gut dysbiosis is a microbial imbalance or maladaptation that activates immunity and promotes metabolic disease. A prebiotic- and fiber-rich diet supports eubiosis, lowers intestinal inflammation and infection risk, and increases short-chain fatty acids (SCFAs), antioxidants, lipid metabolism, and insulin sensitivity. Conversely, excessive sugar, saturated fat, and protein consumption produces a metabolic-syndrome dysbiotic signature: increased Firmicutes/Bacteroidetes ratio, Proteobacteria, Enterobacteriaceae, LPS-producing pathobionts, gut inflammation, cardiovascular risk, permeability, and insulin resistance, alongside reduced SCFAs, Actinobacteria, and SCFA-producer diversity. In the gut-liver axis, fiber-derived acetate, propionate, and butyrate strengthen the barrier, exert anti-inflammatory effects, enhance insulin sensitivity, and regulate lipid metabolism; unmetabolized SCFAs enter the portal circulation. Bile acids activate FXR/TGR5, regulate glucose and lipid metabolism, reshape microbiota composition, and influence inflammation. Barrier dysfunction creates a leaky gut, allowing LPS and other microbial components to translocate into the blood. LPS activates hepatic TLR4/NF-κB signaling, increasing circulating LPS and inflammatory cytokines (TNF-α, IL-6, IL-1β), causing metabolic endotoxemia, hepatic inflammation, steatosis, and insulin resistance. These microbial and metabolic signals are bidirectionally connected: dietary substrate availability shapes community composition, while microbial fermentation products and bile-acid signaling feedback to regulate intestinal immunity, nutrient handling, and host metabolic homeostasis. Reduced barrier integrity further amplifies microbial antigen exposure and inflammatory signaling, linking the intestinal lumen to systemic circulation and hepatic metabolism. The resulting endotoxemic, inflamed, insulin-resistant state integrates the depicted pathway into the development and progression of metabolic syndrome. Thus, diet-induced dysbiosis reduces SCFAs, expands pathobionts, alters bile acids, and disrupts the epithelial barrier; LPS enters the blood, activates the liver, and ultimately promotes metabolic syndrome. Created with BioRender.com.

Its abundance is markedly depleted in individuals with MetS and inversely correlated with inflammatory markers and insulin resistance. *Roseburia intestinalis*, *Anaerostipes* species, and Eubacterium hallii, other major butyrate- and propionate-producing taxa, are also lost in MetS individuals. The depletion of these taxa increases intestinal mucosal permeability by reducing butyrate synthesis and decreasing tight junction protein expression (8, 70, 71). The putative protective role of the mucin-degrading species *Akkermansia muciniphila* of the phylum *Verrucomicrobia*, which enhances intestinal barrier integrity by inducing mucus production and fortification of the mucosal barrier, has been extensively documented in MetS and obesity cohorts, showing an inverse relationship with BMI, plasma glucose levels, and systemic inflammation (72, 73).

### 5.2 Expansion of pro-inflammatory and endotoxin-producing taxa

**Expanding the ‘bad guys’:** The reported loss of ‘good guys’ is complemented by an expansion of ‘bad guys’, particularly pro-inflammatory taxa, in MetS. Many of these reside within the phylum *Proteobacteria* and the Enterobacteriaceae family of Gram-negative species (e.g., *Escherichia*, *Klebsiella*, *Shigella*). These bacteria have LPS-bearing outer membranes and potently activate TLR-4 (74–76).

Enterobacteriaceae expansion is often associated with blood LPS expansion (metabolic endotoxemia), which has been causally linked to systemic inflammation and insulin resistance. Other pathogens enriched in MetS include species in Clostridium cluster IV (e.g., *Ruminococcus gnavus*), which are causally linked to inflammatory bowel disease and mucin degradation, as well as sulfate-reducing bacteria, including Desulfovibrio. These cause epithelial bacterial toxicity and may produce hydrogen sulfide. This expansion of bacterial pathogens, along with a decrease in commensals that produce the beneficial short-chain fatty acid (SCFA), ‘swings’ the balance towards a pro-inflammatory, endotoxin-rich ecosystem that drives both intestinal and systemic inflammation (77, 78) (**Figure 3**).

### 5.3 Functional consequences: metabolic endotoxemia and barrier dysfunction

MetS dysbiosis reflects not only compositional but also functional disruption of the gut microbial ecosystem and its metabolic output, affecting host-microbial interactions in a complex manner (8). Reduced luminal SCFA-producing bacteria and a corresponding decrease in epithelial energy sources (butyrate), tight junction formation, and anti-inflammatory effects promote intestinal epithelial hypoxia and energy depletion, further impairing epithelial growth and promoting intraepithelial cell apoptosis, crypt hyperplasia, and ulceration (42, 57, 79). As a result, intestinal permeability is further heightened, and luminal concentrations of endotoxins (LPS) from Gram-negative bacteria are further increased by the expansion of LPS-producing *Proteobacteria* (reflected by an increase in the prevalence of Enterobacteriaceae); additionally, bacterial product translocation into the portal and systemic circulation is facilitated by the epithelial barrier disruption, which, in turn, increases systemic concentrations of LPS (80–82).

These enterocyte-derived endotoxins induce TLR-4 activation on macrophages, adipocytes, pancreatic beta cells, and hepatocytes, initiating the MyD88-dependent activation of the nuclear factor kappa-B signaling pathway, tumor necrosis factor-alpha, interleukin-6 (IL-6), and interleukin-1 (IL-1)-beta production, which impair insulin signaling by serine phosphorylation of insulin receptor substrate-1 and triggering of c-Jun N-terminal kinase, respectively, providing a mechanistic link of the “gut barrier–endotoxemia–inflammation” axis in the pathogenesis of insulin resistance (83, 84).

## 6 Immunopathological mechanisms linking MetS and gut microbiota

### 6.1 Toll-Like receptor 4 signaling and innate immune activation

TLR-4 is the dominant pattern recognition receptor for LPS and a key driver of immune dysregulation in MetS. In the normal state, TLR-4 signaling in intestinal epithelial cells and innate immune cells induces tolerance to bacteria via strict regulation of its compartmentalization and expression. In MetS, luminal endotoxin load, barrier dysfunction, and circulating free fatty acids, which have also been shown to activate TLR-4, Synergize to promote TLR-4 activation and propagate inflammatory gene-expression cascades (85, 86).

In adipose tissue, TLR-4 activation on macrophages and adipocytes contributes to the recruitment and polarization of classically activated (M1) macrophages that produce tumor necrosis factor-α and IL-6, which coerce neighboring cells to become insulin resistant. In the liver, TLR-4 activation of Kupffer cells induces cytokine expression that promotes hepatic insulin resistance and steatosis. TLR-4 signaling in skeletal muscle induces local inflammation that diminishes glycogen storage and glucose uptake. Systemic TLR-4 activation provides an etiology for the intestine’s central role in cross-organ meta-inflammation and identifies potential therapeutic targets (87–89).

### 6.2 The NLRP3 inflammasome and interleukin-1 beta processing

Metabolic endotoxemia induces not only TLR-4-dependent nuclear factor kappa-B activation, but also the formation and activation of the nucleotide-binding oligomerization domain-like receptor family pyrin domain-containing 3 (NLRP3) inflammasome in macrophages and other innate immune cells (90, 91). The NLRP3 inflammasome (a multimeric protein complex in the cell cytoplasm) ultimately activates pro-caspase-1, which cleaves it to active caspase-1 to allow proteolysis of pro- IL-1-beta and pro- IL-18 into their active, secreted form. Multiple pro-inflammatory signals mediate NLRP3 activation and converge in insulin-resistant states like MetS (92, 93).

LPS primes cells, leading to TLR4-dependent upregulation of NLRP3 and pro-IL-1-beta expression, while metabolic danger signals, including high extracellular adenosine triphosphate, uric acid crystals, mitochondrial reactive oxygen species, and high levels of saturated fatty acids, activate the inflammasome (91, 94).

Active IL-1-beta is one of the most potent pro-inflammatory cytokines and promotes insulin resistance, pancreatic beta-cell death and dysfunction, and immune cell recruitment to metabolic tissues. The NLRP3 inflammasome is activated not only by LPS from the GM, but also *by metabolic changes driven* by altered SCFA and bile acid production (95, 96).

### 6.3 Short-chain fatty acids as immunomodulatory signals

These molecules’ immune-modulating effects provide an essential check against the systemic inflammation seen in MetS. Butyrate, propionate, and acetate exert anti-inflammatory effects by converging on multiple signaling pathways. GPR43 activation on neutrophils and macrophages inhibits inflammatory responses and the generation of reactive oxygen species. Butyrate-driven inhibition of histone deacetylases in T cells dampens inflammation by inducing and expanding regulatory T cells through IL10 and TGFβ production (97, 98).

Butyrate also upregulates tight junction proteins (occludin, claudin-1, and ZO-1), strengthening the intestinal epithelial barrier and limiting paracellular permeability and endotoxin (LPS) translocation. Short-chain fatty acid-producing bacterial genera and metabolite levels decrease in MetS, which alleviates the systemic inflammation-inducing effects of gut microorganisms. Restoring luminal SCFA levels using dietary fiber, prebiotics, or FMT is an emerging approach to reduce systemic inflammation and mitigate MetS (99–101).

### 6.4 Bile acid-mediated immune modulation

Bile acids modulate immune cell activity through signaling via farnesoid X receptor and TGR5. Farnesoid X receptor (FXR, aka NR1H4) is expressed on macrophages, dendritic cells, and T cells. Bile acid activation of FXR inhibits nuclear factor kappa-B signaling and inflammatory cytokine production. TGR5 activation on macrophages reduces inflammation and induces anti-inflammatory M2 macrophage polarization (79, 102, 103).

In MetS, altered microbiota composition and dysbiosis change the bile acid pool: reduced secondary bile acid production activates these anti-inflammatory pathways, and a potential shift toward a greater relative amount of primary bile acids, which have less desirable signaling properties (104). Treatment with the TGR5 agonist obeticholic acid has shown benefits in human metabolic disease, indicating the therapeutic relevance of bile acid signaling in MetS. Thus, microbiota-derived bile acid metabolites that regulate innate immune cell activity may be a modifiable axis connecting microbiome function to systemic inflammatory tone (11, 104).

### 6.5 T regulatory cell and T helper 17 cell axis disruption

Intestinal Barrier and Microbiota-Mediated Control of Homeostasis of Treg/Th17 Axis Among the IL-17-producing cells, T helper 17 cells, or Th17 cells, produce IL -17 and -22 and protect against mucosal pathogens but can promote tissue inflammation, whereas in homeostasis, maintenance of the biological functions of T cells mainly depends on the regulation of Treg cells by IL-10, TGF-β, and other pathways (105–107). Both Th17 cells and Treg cells upregulate the transcription factor ROR, and Treg cell regulation depends on Foxp3 expression. Treg cells also suppress responses to self-antigens and commensal bacteria by promoting IL-10 and TGF production. In MetS, the Treg/Th17 balance shifts toward the pro-inflammatory Th17 side in the gastrointestinal mucosa and visceral adipose tissue (108, 109).

In MetS, multiple microbiota factors shift this balance: cytokines and molecules such as butyrate limit Treg induction and reduce Foxp3 stability; reduced secondary bile acids negatively affect Tregs; and reduced aryl hydrocarbon receptor ligands limit IL-22 production. This Treg loss and Th17 accumulation perpetuate inflammation in the gut, visceral fat, and the liver, ultimately leading to insulin resistance, impaired visceral fat function, and fatty liver characteristic of MetS (110, 111).

### 6.6 Innate lymphoid cells and mucosal immunity

Innate lymphoid cells, particularly group 3 innate lymphoid cells, form a major interface between the GM and adaptive immune responses. ILC3s synthesize IL-22 following sensing of microbial products, such as aryl hydrocarbon receptor (AhR) ligands and IL-23, which enhances epithelial barrier integrity, stimulates antimicrobial peptide production, and modulates microbial composition (112, 113). Dysbiosis and MetS are associated with increased permeability to intestinal contents and decreased IL-22 production, impairing epithelial barrier integrity. The resulting loss of antimicrobial peptides and increased endotoxin levels are further sustained by an expansion of pro-inflammatory bacteria (114, 115). Microbiota-dependent IL-22 production modulates Treg and Th17 cell proliferation and may contribute to the imbalance between these two immune cell populations during MetS. Modulating the microbiota to restore ILC3 activity appears a promising therapeutic approach (116, 117).

### 6.7 Immunoglobulin A and microbial coatings

IgA is the most abundant immunoglobulin isotype at mucosal surfaces, where it promotes mutualistic host-microbe interactions by excluding microbes and regulating bacterial colonization. Dysbiosis correlates with altered IgA coating of gut bacteria in MetS, and changes in the specific bacterial composition targeted by IgA also indicate dysbiosis (118, 119). In murine models, gut IgA+ immune cells regulate obesity-induced insulin resistance, and transferring IgA-targeted microbiota influences the metabolic phenotype. The IgA-microbiota axis therefore appears to be another nuanced mechanism by which immune regulation influences dysbiosis in MetS and its systemic consequences, although the mechanisms of this transfer and the potential for future therapeutic interventions remain poorly understood (120, 121).

## 7 Evidence from animal models

### 7.1 High-fat diet-induced obesity and insulin resistance

Animal models, especially high-fat diet-fed rodents, first showed that GM could cause MetS. In C57BL/6J mice, a high-fat diet induces obesity, insulin resistance, glucose intolerance, and dyslipidemia within weeks, fully recapitulating the MetS phenotype seen in humans. These metabolic disturbances are associated with dramatic changes in the GM (reduced *Bacteroidetes*, expanded *Firmicutes*, and expanded *Proteobacteria*) (122, 123). The ability to transfer the obesity phenotype to germ-free mice transplanted with the GM of obese donors provided irrefutable evidence that the GM causally drives obesity. In addition, transferring microbiota from high-fat diet-fed, insulin-resistant mice to germ-free recipients caused the same phenotype, whereas transferring microbiota from high-fat diet-fed, insulin-resistant mice that remained insulin resistant did not (124, 125).

### 7.2 Toll-like receptor and pattern recognition receptor studies

In microbiota research, genetic models have clarified the necessity of TLR signaling in microbiota-mediated metabolic inflammation. Loss of either TLR4 or its coreceptor CD14 confers resistance to high-fat diet-induced insulin resistance and adipose tissue inflammation, lending support to the so-called ‘metabolic endotoxemia’ hypothesis, but careful interpretation of studies that lack some lines of control for TLR4 deficiency is required because inconsistent results have been reported depending on the host genetic background, the composition of the diet, and the duration of intervention (126, 127).

TLR2-deficient mice that are reared under conventional, but not germ-free, conditions exhibit a dysbiotic microbiota defined by a strong reduction in Bifidobacterium and increased total plasma LPS, with subsequent insulin resistance, glucose intolerance, and obesity, which can be transferred to wild-type germ-free mice by transfer of the microbiota or rescued by antibiotic treatment, indicating an essential role of TLR2 in controlling microbiota composition and subsequent susceptibility to disease. In addition, the tissue-specific role of MyD88, the shared intracellular signal mediator downstream of all TLRs and some IL-1 receptors, was more clearly understood: deletion of MyD88 in hepatocytes causes glucose intolerance and insulin resistance, whereas deletion of MyD88 in intestinal epithelial cells is protective against high-fat diet-induced obesity and insulin resistance (128–130).

### 7.3 Antibiotic manipulation of the gut microbiota

Researchers have also broadly used antibiotics to alter GM and assess the microbiota’s causal role in MetS development in animals (risk phenotype). A six-week intervention of either vancomycin or metronidazole in high-fat diet-fed C57BL/6J mice beneficially modulates the structure of GM and mediates insulin signaling and glucose homeostasis improvement in the ‘obesity-prone’ strains. Interestingly, the metabolic benefits are associated with decreases in inflammatory gene expression in all tested tissues, including the adipose tissue, liver, and the colonic lamina propria; reduction of serum tumor necrosis factor-alpha levels; and rescue of the bile acid receptor TGR5 expression levels in the liver (131–133).

More importantly, the host-genotype-specific benefits are striking: B6J hosts, which are relatively more prone to develop MetS, benefit greatly from antibiotic treatment. In contrast, A/J2 hosts, which are more resistant to insulin resistance due to their unique natural microbiota composition, do not experience metabolic benefits from antibiotic treatment, even though their microbiota composition was similarly altered (134, 135).

These demonstrations clearly indicate that GM affects multiple hosts with diverse genetic backgrounds. Antibiotic treatment’s effects on hosts are not due to the drugs themselves but to modulation of host-microbiota interactions. Furthermore, transferring antibiotic-altered microbiota from donors to recipients can reproduce MetS amelioration in recipients without antibiotic treatment, further supporting the notion that microbiota alteration plays a causative role in MetS across multiple hosts (135–137).

### 7.4 *Akkermansia muciniphila* and beneficial commensals

A mucin degrader, *Akkermansia muciniphila*, is emerging as a particularly promising probiotic species for obesity and other metabolic disorders. In diet-induced obese mice, both live and pasteurized *Akkermansia* have been shown to improve major metabolic markers, including body weight and fat mass gain, fasting blood glucose and insulin, insulin sensitivity, and lipid levels. Studies have shown improved gut barrier integrity, including increased mucus production, increased expression of tight junction proteins, and decreased gut permeability and plasma LPS levels (138, 139). Supplementation with pasteurized *Akkermansia* has been shown to positively affect cytokines and macrophage phenotypes associated with adipose tissue inflammation, including increased levels of regulatory T cell markers and cytokines. Researchers have identified an outer membrane protein as potentially critical to transducing *Akkermansia*’s metabolic benefits through the intestine (140, 141).

### 7.5 Fecal microbiota transplantation studies

FMT in animal models has also yielded definitive proof that metabolic traits are transmissible through GM. Obese microbiota transplanted into germ-free mice results in greater adiposity than germ-free mice receiving microbiota from lean donors, despite similar intakes of calories. Transferring lean microbiota into obese mice improves metabolic deficits. This has been extended to show that defined bacterial strains, or combinations of strains, can reproduce or rescue the phenotype. The colonization of gut-sterile mice with Enterobacter cloacae B29, an endotoxin-producing bacterium isolated from an obese human in an epidemiological case series, was shown to produce obesity and insulin resistance when combined with a high-fat diet (but not normal chow), directly linking a single bacterial pathogen to metabolic pathology (125, 142, 143).

## 8 Evidence from human clinical studies

### 8.1 Cross-sectional and observational studies

Human cross-sectional studies have shown associations between the GM and the MetS components. MetS subjects have lower microbial gene richness, decreased levels of butyrate producers such as Firmicutes, and increased levels of LPS producers such as Proteobacteria than controls; low bacterial gene richness has been associated with adiposity, insulin resistance, and dyslipidemia (144, 145). In people with T2D, gut metagenome analyses have shown enrichment of sulfate-reducing bacteria and decreased butyrate-producing bacteria compared with non-diseased controls, along with decreased butyrate gene/pathway content and increased oxidative stress response gene content. Although these correlational studies did not establish causality, they provided hypotheses for intervention studies to test (146, 147).

### 8.2 Fecal microbiota transplantation in humans

Transplanting microbiota from lean donors to individuals with MetS has provided unequivocal evidence that manipulating the gut microbial community can improve human metabolic health. More specifically, a landmark human randomized controlled trial by Vrieze et al. in which male recipients with MetS received small intestinal infusions of microbiota from lean donors showed significant improvements in peripheral insulin sensitivity 6 weeks post-transplant compared with control recipients who received an autologous microbiota suspension. Insulin sensitivity improvements positively correlated with a higher relative abundance of butyrate-producing bacteria in the recipient microbiota at 6 weeks (148). This study suggests that a global compositional shift in the GM may not drive positive metabolic outcomes, but rather the restoration of specific microbial pathways. However, this technique has several limitations, and more recent reports have shown inconsistent long-term metabolic effects after FMT, though clinical trials are ongoing.

### 8.3 Probiotic and prebiotic interventions

Probiotic supplementation in human MetS interventions has produced mixed results across more than 100 clinical trials. Supplementation with probiotic *Bifidobacterium* and *Lactobacillus* strains improved gut barrier function, systemic inflammatory indices, and moderately improved cardiometabolic parameters such as fasting glucose, insulin sensitivity, and lipid profile, with results varying in strength across trials depending on strain choice, dosage, duration, and baseline gut microbiome composition. Nonetheless, the most convincing human data on MetS-related probiotics come from a trial that supplemented *Akkermansia muciniphila* (149–152).

In a pilot study, 1010-dose daily pasteurized *Akkermansia muciniphila* supplementation for 3 months in adults with MetS was well tolerated and significantly improved insulin sensitivity, insulin levels, and plasma total cholesterol compared with placebo, while live bacteria improved despite trends toward better clinical measures. This difference may relate to the dose of pasteurized bacteria or to the unique accessibility of outer membrane proteins or other antigens on pasteurized bacteria, which may trigger immune stimulation that unlocks metabolic benefits. Later, in our evaluation of *Akkermansia muciniphila* interventions in overweight and obese type 2 diabetics, we also found it to be a successful clinical approach, with benefits primarily observed in those with low baseline fecal *Akkermansia muciniphila* (153–155).

### 8.4 Dietary interventions and microbiota modulation

Among non-invasive methods, dietary intervention can easily modulate GM in MetS. In general, high-fiber diets promote a bloom of short-chain fatty acid-producing bacteria and increase fecal and plasma SCFAs, with glycaemic, satiety signaling, and anti-inflammatory benefits (57, 144). Conversely, Western diets high in saturated fats and refined sugar reduce microbial diversity, decrease Firmicutes, and increase Proteobacteria, with clear increases in inflammation. The typical Mediterranean diet, rich in polyphenols, fiber, and unsaturated fats, is associated with healthier microbial composition and concomitant metabolic improvement in MetS. However, responses to microbiota-targeted dietary interventions show tremendous inter-individual variability, which may predict future interventions and support the development of precision approaches for clinical benefit in humans (156, 157).

## 9 Antibiotic resistance in the context of metabolic dysbiosis

### 9.1 The gut resistome and antimicrobial resistance genes

The gut resistome, sometimes called “the collection of antimicrobial resistance (AMR) genes in the intestinal microbiota,” is the entire collection of AMR genes in the gut microbial community. It includes resistance determinants native to GMs and those acquired via horizontal transfer (158). The human gut resistome is large and can fluctuate widely after antibiotic exposure, dietary changes, and shifts in microbial community structure, which MetS can alter. The composition of the gut microbial community, as well as disease characteristics, may influence the resistome, although interactions between metabolic disease and the extent of resistance genes remain poorly defined. At the population level, antibiotic use has been linked to increased mobile resistance genes, with population antibiotic consumption providing the best predictor of their presence (158, 159).

Firstly, the gut resistome comprises ARGs present in both commensal and pathogenic bacteria inhabiting the gut microenvironment (160). Metagenomic studies have shown that the human gut contains thousands of diverse ARGs that confer resistance to nearly all antibiotics used by humans (161, 162). Although antibiotic use is a major source of selection pressure driving ARG evolution, other factors, such as an individual’s diet, environment, and health status, also shape the nature and abundance of resistome components (163, 164).

The gut is a hotspot for HGT because it has several characteristics, such as high bacterial concentration, biofilm formation, and abundant bacteriophages, which together facilitate the movement of ARG-carrying MGEs between bacteria, across genera, and even among phyla (165, 166). As a result, resistance traits can transfer from harmless commensals to pathogenic bacteria, and vice versa. In particular, commensals can transfer resistance traits to ESKAPE pathogens. The ESKAPE pathogens include *Enterococcus faecium*, *Staphylococcus aureus*, *Klebsiella pneumoniae*, *Acinetobacter baumannii*, *Pseudomonas aeruginosa*, and *Enterobacter* species, and they cause nosocomial infections (167–172).

### 9.2 Antibiotic-induced dysbiosis and metabolic consequences

Both therapeutic and environmental antibiotic exposure can cause major, often persistent changes in microbiota composition similar to the dysbiosis noted in MetS. Even brief antibiotic exposure can deplete microbial diversity, reduce producers of butyrate and other beneficial SCFAs, such as bifidobacteria, and lead to overgrowth of resistant Enterobacteriaceae. Changes in microbiota composition after antibiotic exposure may not be complete, and some taxonomic and functional changes may last for months to years (173, 174).

Alongside the underlying dysbiotic signature in MetS, antibiotic exposure can worsen abnormalities, further impair barrier function, and enhance inflammatory signaling. Pediatric populations exposed to antibiotics in early life have had an increased rate of developing overweight and metabolic disturbances as children, presumably due to a higher rate of microbiota-driven impairment of immune and metabolic programming. In adults, antibiotic therapy has been shown to alter bile acid composition, lower SCFA concentrations, and increase the propensity for metabolic endotoxemia, which can negatively affect adults with existing MetS (175, 176) (**Table 2**).

**Table 2:** Determinants of gut resistome expansion in metabolic syndrome.

Determinant	Effect on resistome	Main mechanism	Clinical/metabolic relevance	Ref.
Western diet	↑ ARG abundance and resistant taxa	High energy density, low fiber, and altered bile-acid/metabolic environment favor resistant pathobionts and reduce ecological competition.	Higher antimicrobial-resistance burden may coexist with obesity, inflammation, and metabolic dysfunction.	(177)
High-fat intake	↑ Mobilome activity and HGT potential	Diet-driven ecological stress and changes in microbial composition can favor mobile genetic elements (MGEs), plasmids, and interbacterial transfer.	This facilitates the acquisition and dissemination of ARGs within an already dysbiotic gut community.	(178)
Low dietary fiber	↑ ARG persistence	Reduced SCFA production and colonization resistance; expansion of facultative anaerobes can create a niche for resistant organisms.	Increases persistence of multidrug-resistant (MDR) organisms and weakens microbiome recovery.	(163)
Gut dysbiosis	↑ ARG reservoir	Loss of beneficial competitors and expansion of ARG-carrying pathobionts increase the density of resistance determinants in the community.	Raises the probability that resistant organisms or genes persist and reach susceptible hosts.	(179)
Chronic inflammation	↑ HGT frequency	Inflammatory stress and reactive oxygen/SOS-related responses can increase bacterial stress adaptation and promote gene mobility; Proteobacteria may expand.	May accelerate horizontal resistance dissemination while reinforcing metabolic inflammation.	(180)
Increased gut permeability	↑ Translocation risk rather than direct ARG selection	Barrier disruption permits greater movement of bacteria, bacterial products, and inflammatory stimuli across the intestinal wall.	Increases risk of extra-intestinal infection and can expose high-risk patients to resistant gut organisms.	(62)
Obesity and MetS	↑ Enrichment of MDR-associated taxa	Altered diet, inflammation, bile-acid signaling, comorbidity, and medication exposure reshape the microbial ecology toward resistant taxa.	Increases susceptibility to MDR colonization and complicates infection management.	(164)
Antibiotic exposure	Strong and direct ARG selection pressure	Depletes susceptible competitors while resistant organisms survive; reduced diversity can enable long-term persistence of ARG-carrying taxa.	Major driver of long-term resistome expansion and recurrent MDR colonization.	(181)

ARG, antibiotic resistance gene; HGT, horizontal gene transfer; MGE, mobile genetic element; MDR, multidrug-resistant; MetS, metabolic syndrome; SCFA, short-chain fatty acid.

If these individuals harbor a GMM containing multidrug-resistant (MDR) bacteria, their risk of developing life-threatening infections increases greatly. Studies suggest that carriage of MDR microorganisms, such as carbapenem-resistant Enterobacterales (CRE) and vancomycin-resistant Enterococci (VRE), strongly increases infection risk, especially among hospitalized and immunocompromised patients (182, 183). Gut dysbiosis in MetS subjects, characterized by weakened barrier integrity and a pro-inflammatory state, promotes the migration of these MDR pathogens into the blood, leading to sepsis. This syndemic condition of interaction between metabolic disturbances and AMR represents a serious public health threat and requires approaches that account for both aspects (163).

Sepsis is best framed as an immunometabolic emergency: pathogen- and damage-associated signals rewire leukocyte metabolism and antigen presentation, tipping some patients toward hyperinflammation and others toward prolonged immune paralysis. In that setting, infection with multidrug-resistant organisms is less likely to be a direct “autoimmune trigger” by virtue of resistance than a marker of preceding antibiotic pressure, epithelial/mucosal barrier injury, microbiome disruption, endotoxin exposure, repeated hospital-associated immune stimulation, and delayed or suboptimal source control (184). Those conditions can promote molecular mimicry, epitope spreading, superantigen-like T-cell activation, B-cell survival, type-I interferon/IL-6/IL-17 signals, and loss of regulatory control, all plausible contributors to flares in autoantibody-driven diseases such as myasthenia gravis (AchR/MuSK/LRP4) (185). For Myasthenia gravis (MG) specifically, an intercurrent resistant infection or sepsis may amplify thymic/autoimmune B-cell activity, complement, and neuromuscular junction vulnerability, while antibiotics, steroids, IVIG/plasma exchange, nutritional stress, and sedative/paralytic exposure further confound the clinical picture. Thus, the prudent claim is associational and mechanistic rather than proven causation: resistant-microorganism exposure often clusters with the immunometabolic and dysbiotic milieu that can precipitate or worsen autoimmune activity, and MG patients should have infections treated early, antibiotics narrowed when possible, and disease status monitored closely during and after sepsis (184, 186).

### 9.3 Host-dependent effects of antibiotics on metabolism

The effects of antibiotic use on metabolism depend on host genetic background, baseline microbiota, and the antibiotic used. For antibiotics, host genotype, diet, and antibiotic type can produce a range of effects. For example, low-dose penicillin treatment in neonatal mice can modulate metabolism, increasing fat mass and altering fat distribution profiles that can be transferred to germ-free recipients via microbiota transplants and worsened by subsequent high-fat diet feeding (187–189).

In contrast, high-dose antibiotic use in adult mice with a pre-existing obese phenotype can improve some aspects of obesity-related phenotypes by grossly suppressing microbial load and LPS levels. This seems paradoxical and shows that dose and use circumstances can produce vastly different effects; vancomycin treatment in humans has been associated with reduced insulin sensitivity. Loss of SCFA-producing bacteria and reduced glucagon-like peptide-1-dependent metabolic effects may explain the changes observed (189, 190).

### 9.4 Horizontal gene transfer and resistome dynamics

The gut resistome is a major hub for horizontal transfer of resistance genes because of its high bacterial load, biofilm production, and the presence of mobile genetic elements (plasmids, transposons, integrons). Antibiotic administration selectively expands resistant bacteria and promotes horizontal transfer of resistance genes between pathogenic and commensal bacteria (161, 191). In MetS, extreme shifts in gut pH, redox potential, and overall microbial community composition may facilitate or hinder the long-term dissemination of resistance genes. In particular, the expansion of Proteobacteria, known to be effective donors or recipients of resistance genes, may further increase horizontal gene transfer and expand the resistome. Understanding the ecological and evolutionary dynamics of the gut resistome in metabolic disease can help predict the long-term impact of antibiotic use in this setting (192, 193) (**Figure 4**).

**Figure 4:**
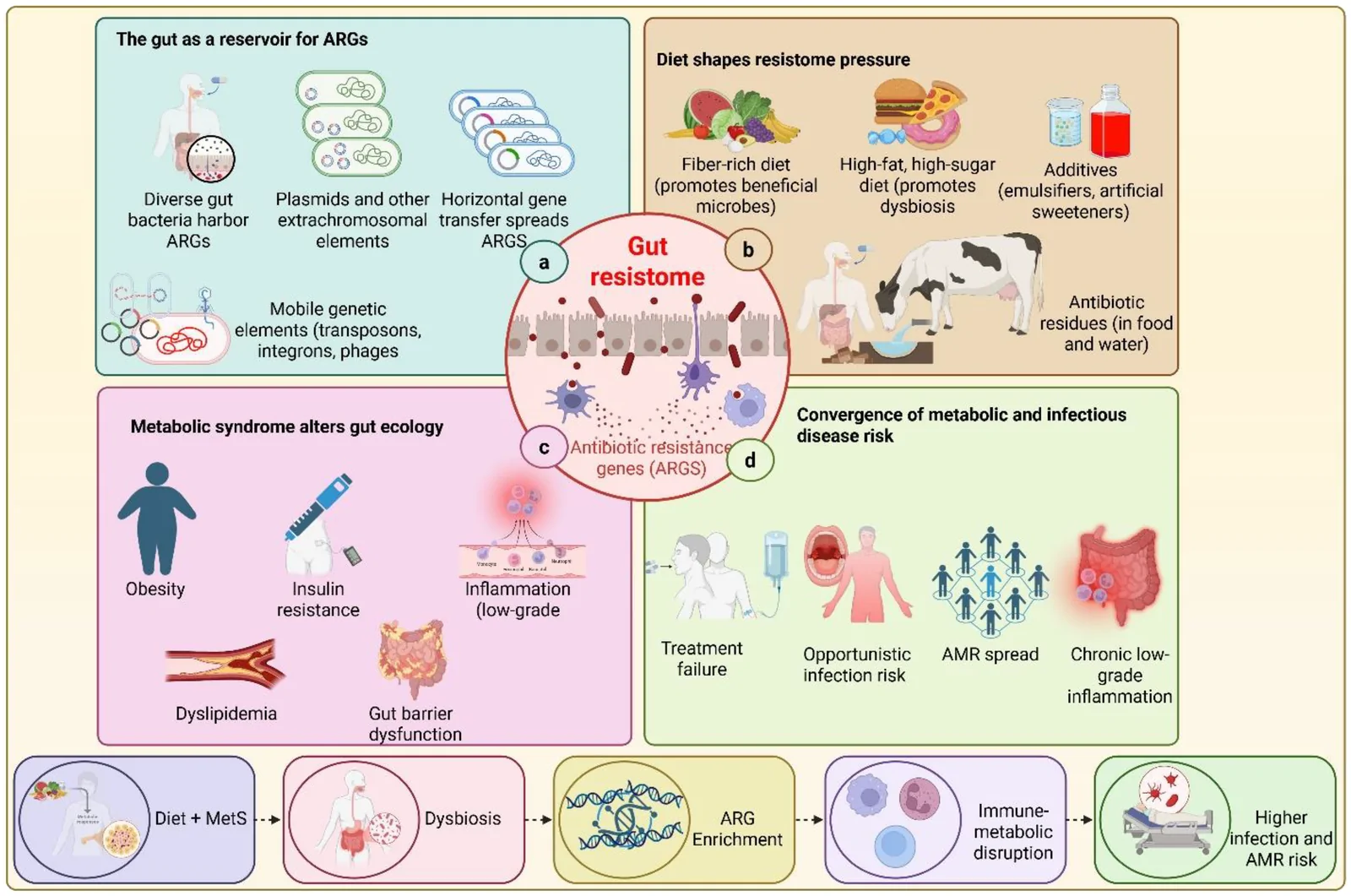
Interactions among gut resistome, diet, metabolic syndrome (MetS), and antimicrobial resistance risk. The central gut-resistome network links all four domains and illustrates their bidirectional interactions and their potential feedback on host metabolic health. **(a)** The gut serves as a reservoir of ARGs carried by diverse bacteria, plasmids, and other extrachromosomal elements, as well as mobile genetic elements—including transposons, integrons, and phages—that promote horizontal ARG transfer. **(b)** Diet shapes resistome pressure: fiber-rich foods support beneficial microbes, whereas high-fat/high-sugar diets promote dysbiosis; food and water may also introduce antibiotic residues, while additives can further influence microbial ecology. **(c)** MetS-associated obesity, insulin resistance, dyslipidemia, low-grade inflammation, and gut-barrier dysfunction intensify ecological disruption and increase ARG enrichment. **(c)** These processes converge on immune-metabolic dysfunction, treatment failure, opportunistic infections, AMR dissemination, and persistent inflammation. Created with BioRender.com.

### 9.5 Clinical implications and stewardship

Implications for Management: The close interplay between MetS, gut dysbiosis, and antibiotic resistance has major implications for managing infections in people with MetS. MetS predisposes people to infections that require antibiotics, while their dysbiotic microbiota predisposes them to developing resistance during antibiotic therapy (20, 163). Appropriate antibiotic stewardship, including avoiding unnecessary antibiotic courses, using narrower-spectrum agents when feasible, and limiting therapy to the shortest duration needed to treat the infection while ensuring resolution, may help restrict resistome expansion in people with MetS. Antibiotics that spare the microbiota, probiotic therapies, and post-therapy microbiome restoration are currently areas of exciting translational research (194, 195).

## 10 Therapeutic implications and microbiota-targeted interventions

### 10.1 Probiotics, prebiotics, and synbiotics

Dysbiosis in MetS, marked by low SCFA, high LPS burden, and loss of barrier-supportive taxa, is a modifiable risk factor that microbiota-modifying interventions may target. Several clinical studies have used probiotic supplementation with *Bifidobacterium* and *Lactobacillus* species, with minor benefits for MetS, including improved markers of inflammation, gut barrier function, and cardiometabolic risk factors. In conventional probiotics, modest clinical effects, significant heterogeneity in colonization efficacy, and the short duration of most trials may limit their utility in treating MetS (8, 196).

Inulin, oligofructose, and resistant starch supplementation, prebiotics that selectively foster growth of SCFA-producing bacteria, have been shown to increase butyrate and propionate concentrations in human feces, as well as improve satiety and glycemic control among people with MetS. Synbiotics, which combine probiotic strains and prebiotics, may optimize probiotic colonization and metabolism, although human MetS trials are limited (197, 198).

### 10.2 Fecal microbiota transplantation and defined consortia

FMT is the most powerful microbiome modulator because it can convert the entire gut microbial ecosystem, and it is already an established treatment modality for recurrent *C. difficile* infection (199). FMT for MetS is at present experimental; however, the upcoming definitive evidence of concept for its efficacy in that context has prompted further interest in microbiome transplantation in the research setting at present, intending to define the most efficacious preparation, standardized donor screening, delivery protocols, predictive response biomarkers, and long-term safety data. Eventually, a more refined and predictable intervention might be possible, using defined bacterial consortia selected *in silico* for the ability to restore specific metabolic functions (199–201).

### 10.3 Postbiotics and microbial metabolites

Postbiotics, biological products that are not live bacteria, are an attractive emerging therapeutic approach. Pasteurized *Akkermansia muciniphila*, which delivers biological benefits despite bacterial inactivation, exemplifies the postbiotic approach. Clinical studies have tested oral or rectal administration of fermentates containing SCFAs (particularly butyrate), bile acid derivatives, tryptophan metabolites, and other bacterial signaling molecules to improve barrier function and counteract inflammation; results in MetS are not yet known (140, 202).

Postbiotic approaches also include targeted delivery of single microbial products, such as bile acids and tryptophan metabolites, and may not require live bacteria. Postbiotics are more stable and standardizable and have a better safety profile than live biotherapeutics, but the functional complexity of microbial metabolic networks and community dynamics may, in some instances, challenge the efficacy of supplementing with a single metabolite (203, 204) (**Table 3****).**

**Table 3:** Microbiota-targeted therapeutic strategies for metabolic syndrome.

Intervention	Examples/forms	Primary microbiome action	Expected metabolic benefit	Resistome considerations	Ref.
High-fiber diets	Whole grains, legumes, vegetables, resistant starch	Provides fermentable substrates that support SCFA-producing bacteria, cross-feeding, and microbial diversity.	Improved insulin sensitivity, better glycemic regulation, stronger epithelial barrier, and reduced inflammatory tone.	May decrease ARG abundance indirectly by restoring ecological competition and SCFA-mediated colonization resistance.	(164)
Mediterranean diet	Fruits, vegetables, whole grains, olive oil, nuts, polyphenol-rich foods	Increases substrate diversity, microbial richness, and SCFA-producing populations while reducing an energy-dense dietary pattern.	Improved metabolic profile and reduced cardiometabolic risk when sustained as a dietary pattern.	May reduce ARG burden by improving community stability and lowering selective pressure from dysbiosis-associated conditions.	(205)
Prebiotics	Inulin, FOS, GOS, and related fermentable substrates	Selectively stimulates beneficial bacteria and promotes cross-feeding and SCFA formation.	Potential improvement in glycemic control, lipid handling, satiety signaling, and barrier function.	May reduce ARG-carrying pathobionts by strengthening colonization resistance; effects depend on substrate and host microbiota.	(206)
Probiotics	*Lactobacillus* and *Bifidobacterium* spp.	Competitive exclusion, metabolite production, and support of epithelial and immune homeostasis.	Usually modest and strain-specific improvements in glucose or lipid metabolism and inflammatory markers.	Effects vary; assess strain safety, transferable ARGs, and persistence rather than assuming a uniformly favorable resistome effect.	(207)
Synbiotics	Probiotic + prebiotic combinations	Combines delivery of selected microorganisms with a substrate intended to improve their survival, activity, and colonization.	May provide broader metabolic improvements than probiotics alone in some settings.	May favorably modulate the resistome through improved community structure, but outcomes remain formulation- and strain-dependent.	(208)
Postbiotics	SCFAs, inactivated cells, microbial metabolites or components	Delivers bioactive microbial products without requiring viable bacteria to establish colonization.	Supports barrier function and may reduce inflammatory signaling and metabolic endotoxemia.	No risk of transfer of live bacterial ARGs from the administered product; manufacturing quality and bioactivity remain important.	(209)
Fecal microbiota transplantation (FMT)	Screened healthy-donor microbiota	Rapidly introduces a complex microbial community and can restore ecological diversity and colonization resistance.	Some patients have shown improved insulin sensitivity, but responses vary and depend on context.	May reduce ARG burden in selected settings, but donor-derived AMR transmission is a major safety issue requiring stringent screening and monitoring.	(210)
Live biotherapeutic products (LBPs)	Defined or engineered bacterial consortia	Precision microbiome modulation using selected strains with defined functions and controlled composition.	Emerging potential for targeted improvement of metabolic and inflammatory pathways.	Potentially lower ARG risk than complex microbiota transfer when strains are genetically characterized and appropriately selected; requires rigorous safety testing.	(211)

FOS, fructooligosaccharides; GOS, galactooligosaccharides; SCFAs, short-chain fatty acids; ARG, antibiotic resistance gene; AMR, antimicrobial resistance; FMT, fecal microbiota transplantation; LBPs, live biotherapeutic products.

### 10.4 Precision microbiome medicine

Future of microbiota-directed therapy in MetS microbiota-oriented therapies The heterogeneity of baseline microbiota composition, genetic background, diet, and metabolic phenotype in MetS requires a personalized approach, which multi-omics integration of metagenomic, metabolomic, transcriptomic, and immunophenotypic data could achieve. Multi-omics analysis of the GM and metabolome may stratify a MetS cohort into microbiota-metabolic subtypes, identifying responders and non-responders to specific interventions (204, 212). Stratification by baseline *Akkermansia muciniphila* and butyrate-producer levels or bile acid homeostasis may support individualized administration of probiotics, prebiotics, and postbiotics. Microbiome-based markers for metabolic risk prediction and treatment monitoring are under development and could enable a personalized, microbiome-guided approach to MetS management (213–215).

## 11 Discussion

### 11.1 Synthesis of immunopathological mechanisms

This review has explored how MetS-related changes in the GM and subsequent changes in microbiota composition and function that drive metabolic and inflammatory dysfunction may be related. Data now indicate that the metabolic and inflammatory states characteristic of MetS (hyperglycemia, increased free fatty acids, and systemic low-grade inflammation) create a permissive inflammatory intestinal luminal environment that is less hospitable to beneficial SCFA-producing commensals and more hospitable to pro-inflammatory, LPS-shedding *Proteobacteria*. Such a compositional shift alleviates SCFA -imparted support of the intestinal barrier and immune-mitigating responses while elevating the intestinal and systemic endotoxin burden, thereby triggering TLR-4 and nucleotide-binding oligomerization domain-like receptor family pyrin domain-containing 3 inflammasome signaling in the metabolic tissues to propagate the production of tumor necrosis factor-alpha, IL-6, and IL-1-beta that adversely affect insulin signaling. Concurrent dysregulation of bile acid metabolism, tryptophan metabolism, and Treg/Th17 immunity would further elevate inflammatory signaling and create a self-reinforcing dysbiotic/meta-inflammatory loop.

### 11.2 Integration of animal and human evidence

Human findings that support and extend the animal data further strengthen the biological plausibility of the microbiota-immunity-metabolism axis in MetS. Animal studies have played a pivotal role in establishing causality through germ-free colonization, FMT, genetic knockout models, and antibiotic-mediated microbiota manipulation, showing that microbiota alterations are not merely an epiphenomenon of metabolic disease but actively contribute to its etiology. Human studies, by their nature, cannot demonstrate causality, but they have established the associations seen in animals and contributed to a translational program using fecal transplantation and probiotic administration. The successes of human FGID trials and the recent proof of concept for microbiota-targeted therapy for MetS by fecal transplantation of lean donor microbiota in MetS patients are remarkable achievements. However, the ultimate aim of implementing microbiota modulation as a standard therapy remains elusive because microbiota composition, immune system structure, and physiology differ by species (216–218).

### 11.3 Assessment of conflicting evidence

Careful consideration is needed for numerous areas of apparent contradiction. The metabolic phenotype of TLR-4 knockout mice is inconsistent: some studies show protection from HFD-induced insulin resistance, while others detect no differences in glucose handling. These disparities may reflect differences in genetic background, diet composition, high-fat diet duration, and tissue-specific TLR-4 deletion (219).

Likewise, antibiotic effects depend on context: low-dose exposure early in life leads to obesity in both animal models and human epidemiological studies, whereas high-dose antibiotic treatment in adults with pre-existing metabolic dysregulation can paradoxically improve metabolism. These marked differences may reflect dose, host genetics, and microbiota composition. Probiotics’ effect on MetS is also highly variable, with some data showing metabolic improvements and other data showing no effect. These findings may reflect variation in probiotic strains, doses, and viability, as well as their ability to colonize the gut (188, 220).

### 11.4 Antibiotic resistance as a metabolic modifier

Incorporating the resistance component into the microbiota-MetS paradigm adds clinically and public-health-relevant features. Antibiotic exposure is almost inevitable in individuals with MetS, who are potentially more prone to infection; this may deepen MetS-associated dysbiosis, reduce taxonomic and functional richness, and enhance selection for resistant strains. The chronic impact of antibiotic exposure on microbiota-MetS may include sustained metabolic disruption through effects on energy extraction, barrier integrity, and immune modulation that persist beyond drug exposure. Additionally, the resistance profile itself can affect metabolic health through resistance independent of antibiotic effect (i.e., Resistant strains could differ in their ability to extract nutrients or induce immune responses compared with sensitive bacteria). Developing microbiota-sparing antimicrobial regimens and post-treatment probiotics seems a worthy goal in the MetS population (221) (**Table 4****).**

**Table 4:** Proposed mechanisms linking gut dysbiosis, metabolic syndrome, and antibiotic resistance.

Dysbiosis change	Key molecular/host pathway	Effect on metabolic syndrome	Effect on antibiotic resistance	Ref.
Increased *Firmicutes*/*Bacteroidetes* ratio	Altered carbohydrate breakdown, acetate production, and lipogenic signaling; ecological shift may also change substrate availability for resistant taxa.	May promote adiposity, weight gain, insulin resistance, and dyslipidemia when part of broader dysbiosis.	Firmicutes-enriched communities may contain abundant tetracycline (tetW) and macrolide (ermB) resistance genes; transposase activity can support horizontal gene transfer.	(222–224)
Reduced *Akkermansia muciniphila*	Thinner mucus layer, altered tight-junction expression, increased permeability, and greater LPS exposure.	Supports low-grade endotoxemia and chronic inflammation and is associated with obesity and insulin resistance.	Loss of a barrier-supporting taxon may permit expansion of Proteobacteria carrying ESBL/carbapenemase genes; preclinical supplementation studies suggest reduced ARG burden.	(155, 225–227)
Loss of butyrate-producing bacteria (e.g., *Faecalibacterium prausnitzii*, *Roseburia*)	↓ Butyrate weakens colonocyte energy supply, epithelial hypoxia, tight-junction support, and anti-inflammatory signaling; increased mucosal oxygen/nitrate can favor Enterobacteriaceae.	Impaired glucose tolerance, hepatic steatosis, and heightened systemic inflammation.	Reduced butyrate may facilitate conjugative transfer of IncF/IncI plasmids and dissemination of ARGs such as blaCTX-M and mcr-1.	(40, 228–230)
Expansion of *Enterobacteriaceae*/*Proteobacteria* with elevated LPS	Higher LPS/flagellin exposure activates TLR4/NF-κB signaling and promotes systemic inflammatory responses.	Metabolic endotoxemia contributes to insulin resistance, adipose inflammation, and fatty liver disease.	Enterobacteriaceae can serve as reservoirs for plasmid-borne ARGs (e.g., blaNDM, mcr-1, qnr); inflammatory stress may favor conjugative transfer.	(223, 231, 232)
Antibiotic-induced loss of microbial diversity	Depletion of competing commensals and disruption of SCFA and bile-acid metabolism alter host and microbial stress responses.	Repeated or early-life antibiotic exposure has been associated with increased adiposity and impaired glucose homeostasis in observational and experimental settings.	Direct selection enriches resistant organisms such as VRE and ESBL-producers; community disruption can increase persistence and promote SOS-associated gene transfer.	(223, 233)
High-fat diet-driven increase in deoxycholic acid (DCA)	DCA can increase oxidative stress, DNA damage, and bacterial SOS responses while also promoting mucosal inflammation.	May amplify hepatic inflammation, insulin resistance, and steatotic liver disease/NASH.	SOS-associated responses can increase integron/integrase activity and facilitate acquisition or spread of ARGs such as sul1 and dfrA.	(234, 235)
Overgrowth of TMA-producing bacteria	Microbial conversion of dietary choline/carnitine increases trimethylamine, which is converted to TMAO; TMAO affects platelet reactivity, lipid handling, and vascular signaling.	Associated with atherosclerotic and cardiometabolic risk, including impaired insulin sensitivity in susceptible settings.	TMA-producing taxa may also harbor multidrug-resistance determinants; choline-rich dietary environments can co-select ecological traits associated with an expanded resistome.	(232, 236)

ARG, antibiotic resistance gene; DCA, deoxycholic acid; ESBL, extended-spectrum β-lactamase; HGT, horizontal gene transfer; LPS, lipopolysaccharide; NASH, non-alcoholic steatohepatitis; NF-κB, nuclear factor kappa B; TLR4, Toll-like receptor 4; TMA, trimethylamine; TMAO, trimethylamine N-oxide; VRE, vancomycin-resistant Enterococcus.

### 11.5 Limitations of current evidence

Most human microbiota studies to date have been either purely observational or based on short-term interventional trials limited by small sample sizes. High inter-individual variability in human GM composition makes it difficult to identify universally applicable signatures of GM dysbiosis. Variability between methods for assessing microbiota (DNA extraction techniques, sequencing platforms, bioinformatics pipelines, and metabolite-measurement techniques) also remains an issue. While many animal models have been used successfully to study the role of microbiota in MetS pathogenesis, none replicate human MetS exactly in terms of its causes, progression, and microbiota composition. The complexity of MetS suggests that targeting the microbiota alone will not reverse all manifestations of MetS without concomitant dietary/lifestyle/pharmacological therapies.

### 11.6 Future directions

For therapeutic interventions to succeed widely, future studies should include large-scale, long-duration randomized controlled trials of microbiota-focused therapies in well-phenotyped MetS groups, with clinically meaningful endpoints including measures of glucolipid metabolism, adiposity and body composition, cardiovascular events, and surrogate markers of cardiovascular disease. Adopting multi-omics technologies, metagenomics, metabolomics, transcriptomics, and immune profiling, will provide mechanistic insights and improve stratification of treatment responders and non-responders. Novel therapeutics, such as defined bacterial consortia and novel postbiotic compounds, may offer more standardized options than fecal microbial transplants. Research on the gut resistome, the effects of antibiotic perturbation on gut microbial networks, and potential effects on the host metabolome is ripe for translation. A precision medicine approach to gut microbiome management should address the heterogeneity that has hamstrung the efficacy of broadly applied probiotic treatments.

## 12 Conclusions

Immunopathology of MetS is inseparable from changes in microbiota composition and function, creating a complex, bidirectional pathogenic cycle that drives systemic inflammation and metabolic disturbances. As with MetS features in the microbiota (depletion of SCFA producers and proliferation of LPS-producing Proteobacteria), dysbiosis likely drives the initial events of MetS, resulting in increased intestinal permeability, inflammatory TLR-4 signaling, and metabolic endotoxemia. Meanwhile, intestinal dysbiosis-induced alterations in microbial bile acid metabolism and tryptophan catabolism, along with a disturbed Treg/Th17 balance, further aggravate the inflammatory response in metabolic tissues. Representative evidence from animal models and clinical trials also supports the causality of these mechanisms and the efficacy of microbiota-based therapies, such as FMT, probiotics, and diet, in MetS. Increasingly, evidence (including the unfavorable effects of antibiotics and antibiotic-associated dysbiosis) shows potential adverse effects of microbiota-targeted interventions on MetS, supporting the development of precision intervention strategies. Technological advances can optimize microbiome-targeted interventions in MetS by translating pathogenic knowledge into clinical solutions.
